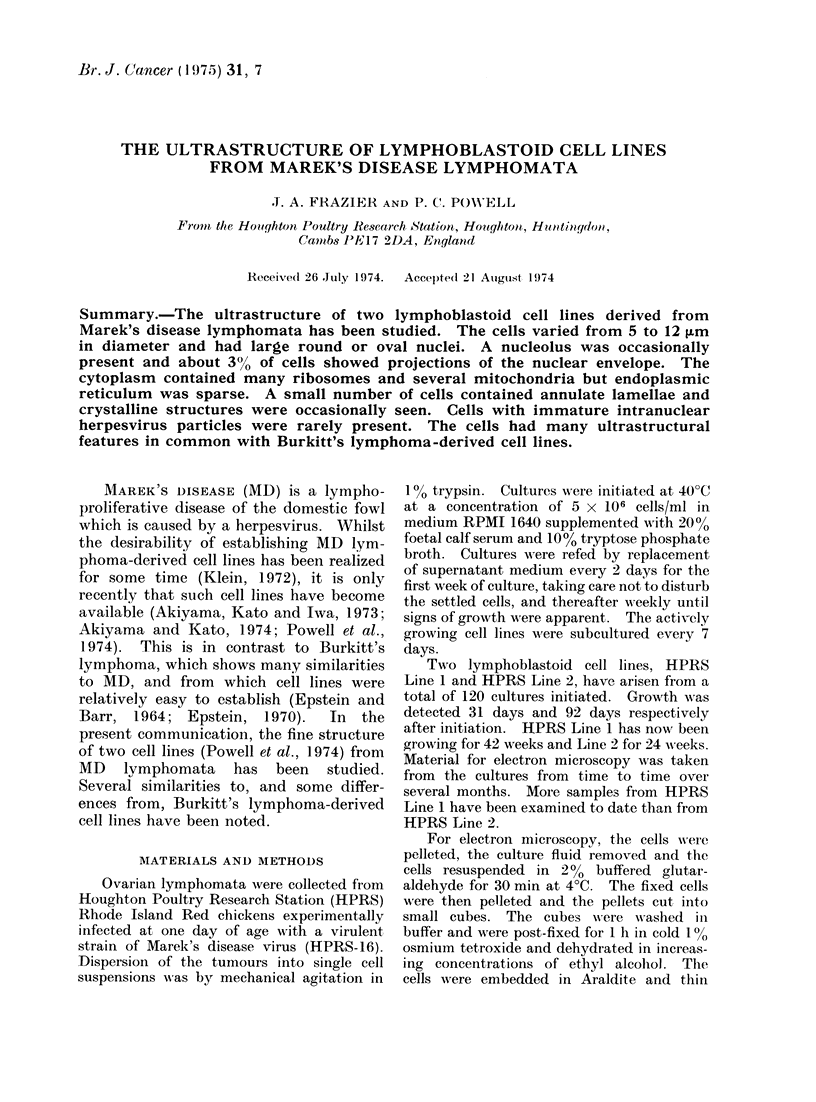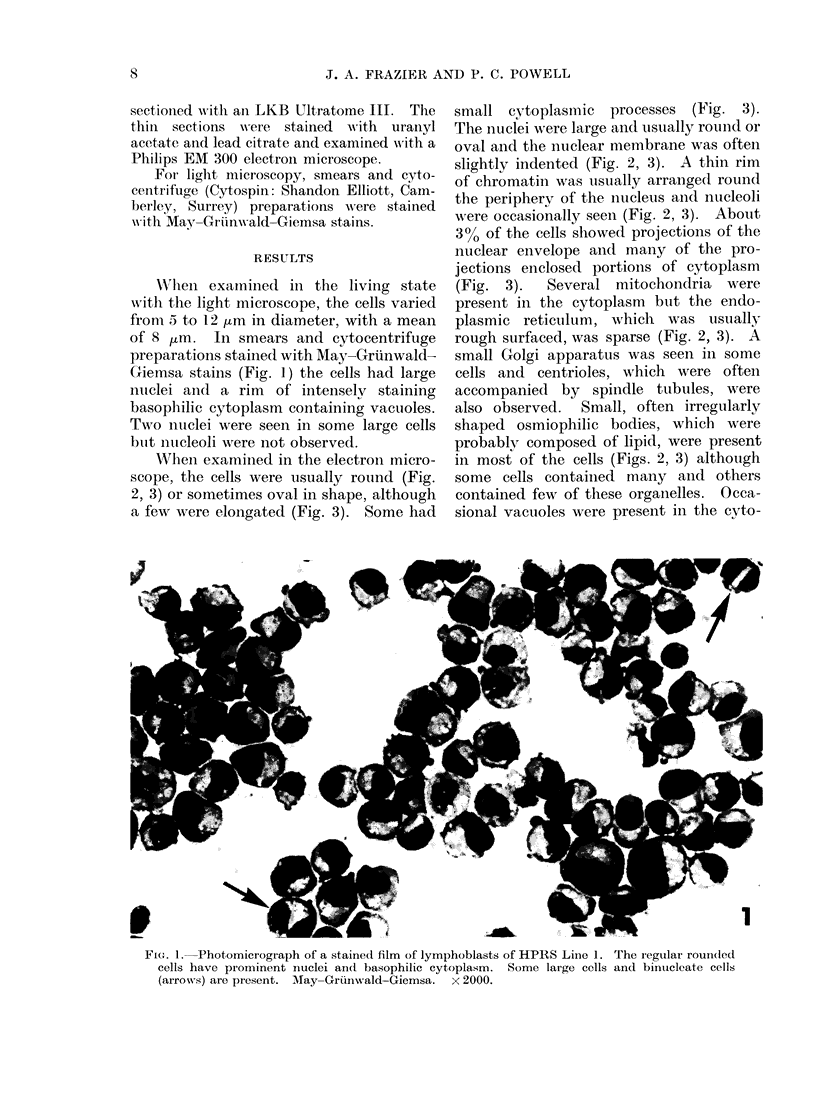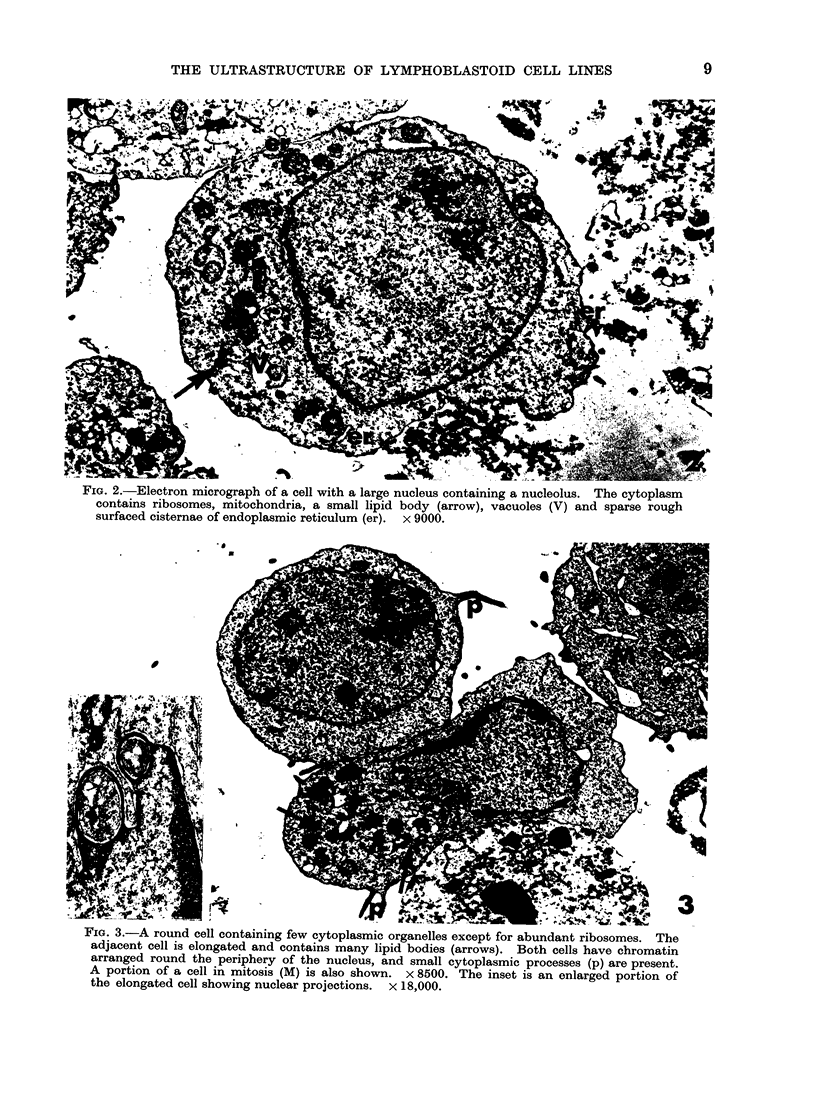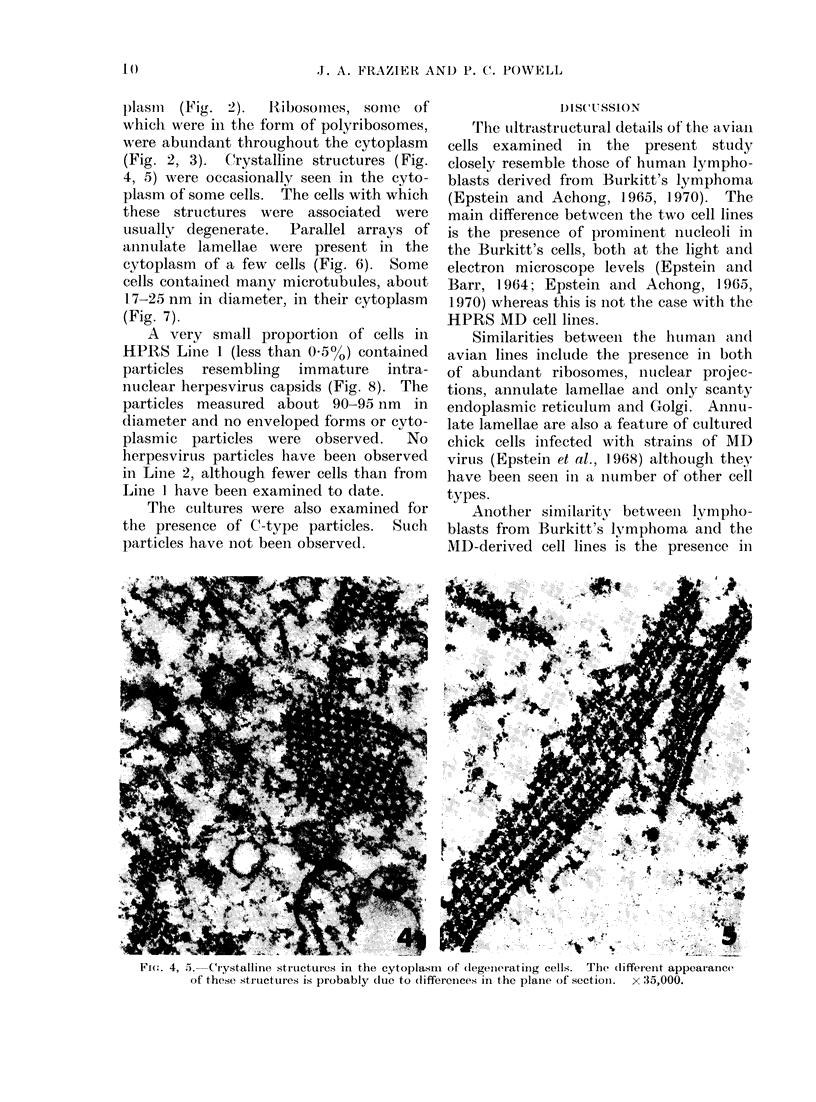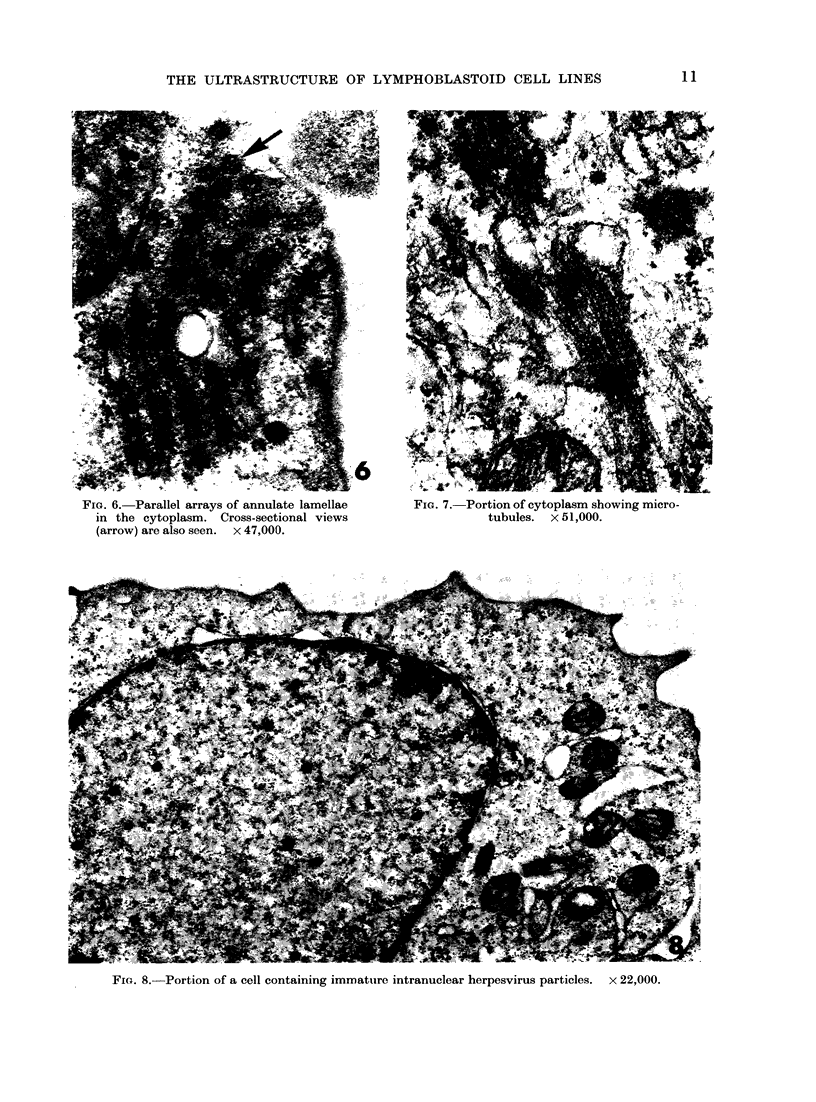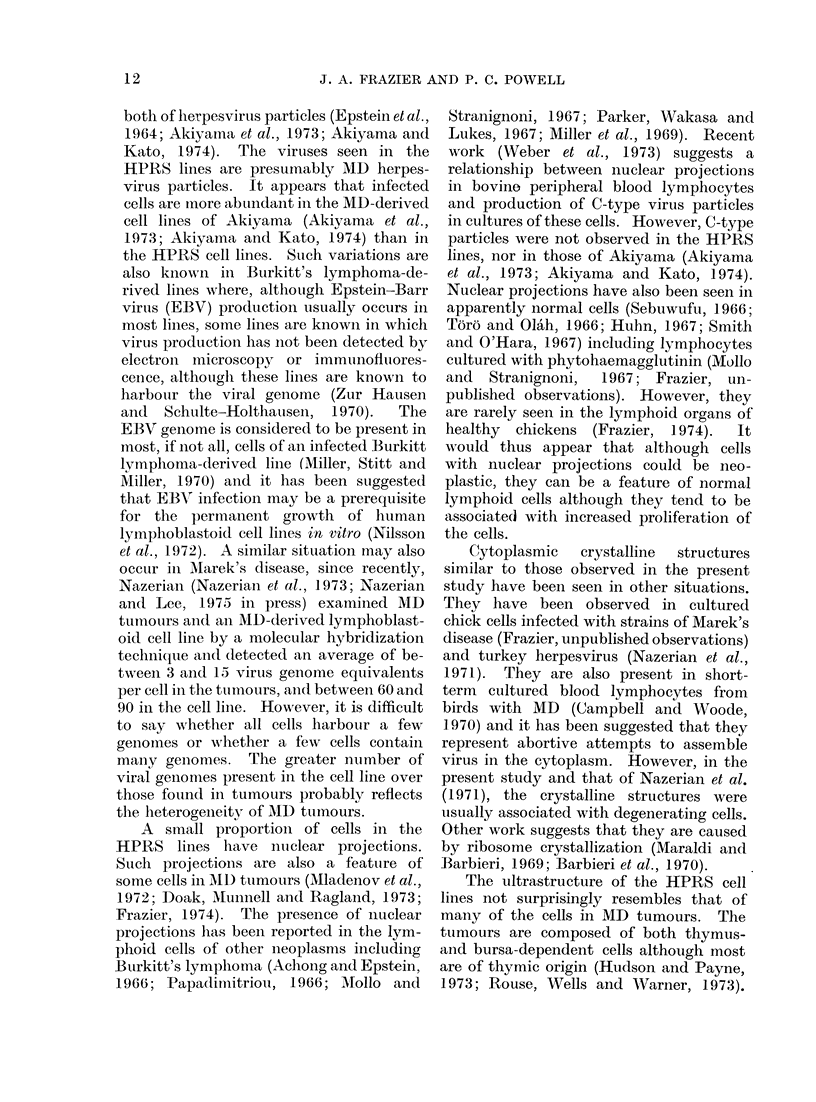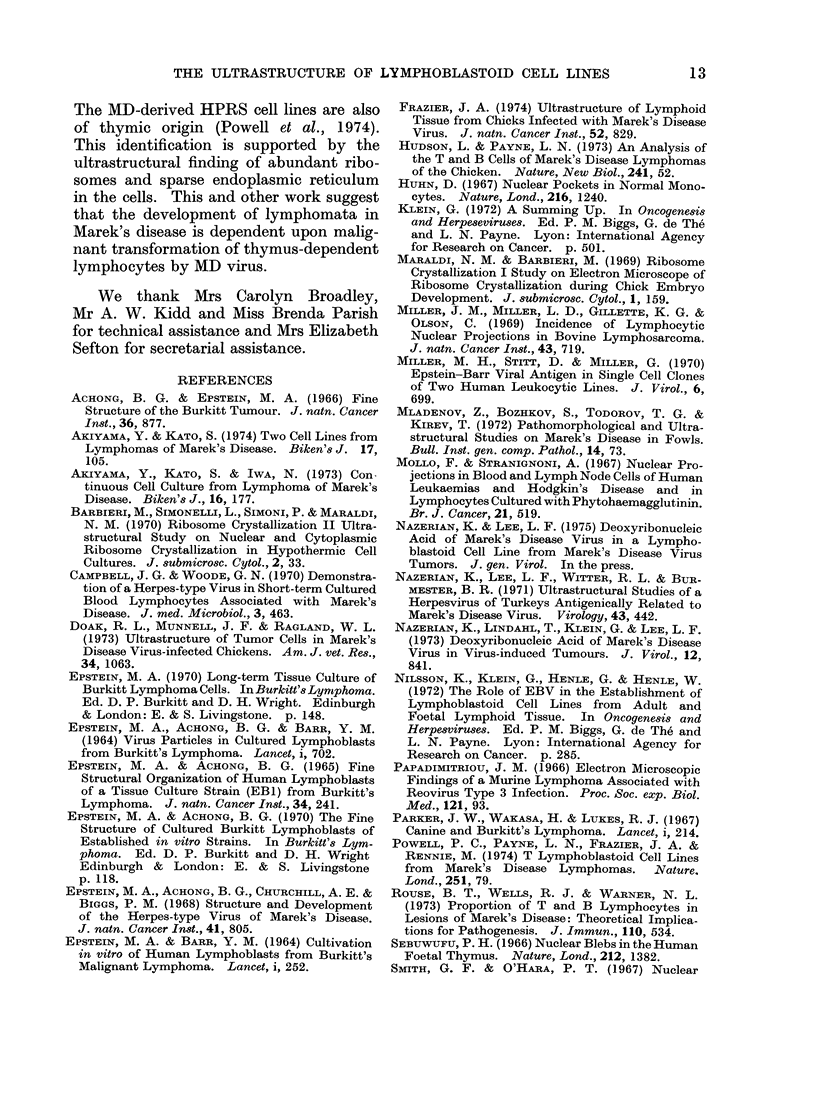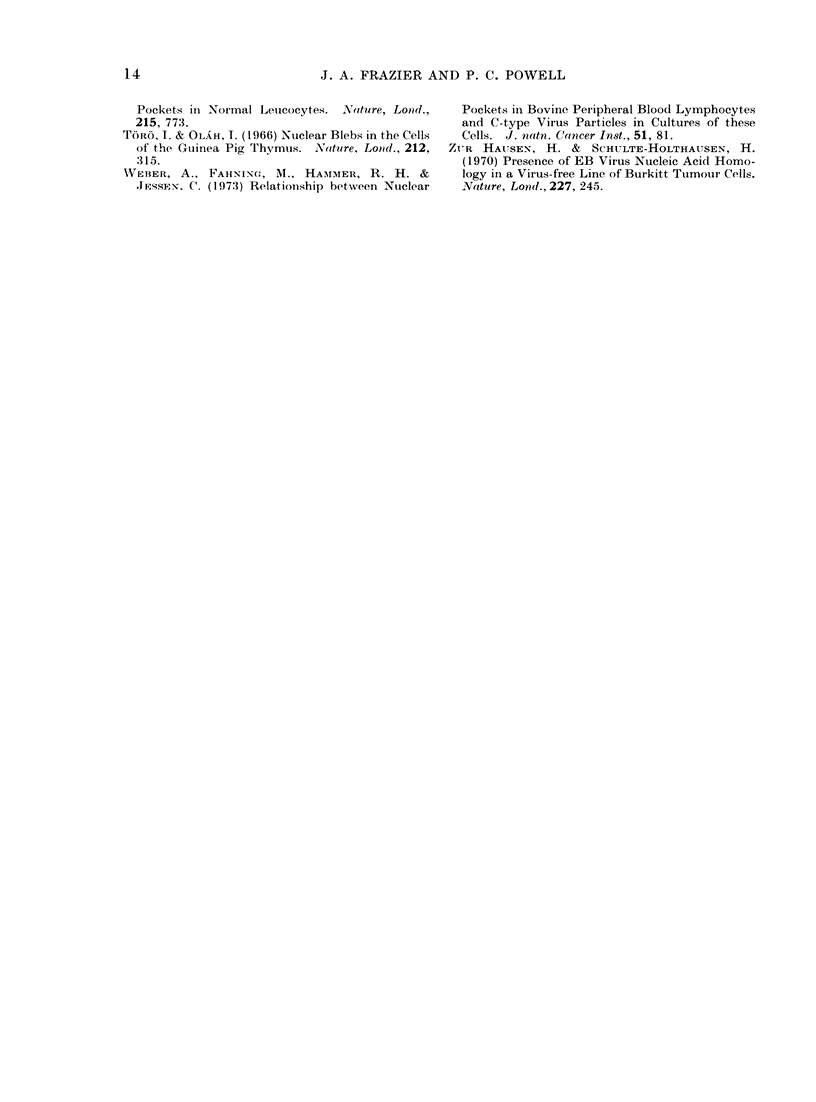# The ultrastructure of lymphoblastoid cell lines from Marek's disease lymphomata.

**DOI:** 10.1038/bjc.1975.2

**Published:** 1975-01

**Authors:** J. A. Frazier, P. C. Powell

## Abstract

**Images:**


*Br. J. Cancer* (1975) **31**, 7

### THE ULTRASTRUCTURE OF LYMPHOBLASTOID CELL LINES FROM MAREK'S DISEASE LYMPHOMATA

J. A. FRAZIER AND P. C. POWELL

*From the Houghton Poultry Research Station, Houghton, Huntingdon, Cambs PE17 2DA, England*

Received 26 July 1974. Accepted 21 August 1974

**Summary.—The ultrastructure of two lymphoblastoid cell lines derived from Marek's disease lymphomata has been studied. The cells varied from 5 to 12 μm in diameter and had large round or oval nuclei. A nucleolus was occasionally present and about 3% of cells showed projections of the nuclear envelope. The cytoplasm contained many ribosomes and several mitochondria but endoplasmic reticulum was sparse. A small number of cells contained annulate lamellae and crystalline structures were occasionally seen. Cells with immature intranuclear herpesvirus particles were rarely present. The cells had many ultrastructural features in common with Burkitt's lymphoma-derived cell lines.**

MAREK'S DISEASE (MD) is a lymphoproliferative disease of the domestic fowl which is caused by a herpesvirus. Whilst the desirability of establishing MD lymphoma-derived cell lines has been realized for some time (Klein, 1972), it is only recently that such cell lines have become available (Akiyama, Kato and Iwa, 1973; Akiyama and Kato, 1974; Powell *et al.*, 1974). This is in contrast to Burkitt's lymphoma, which shows many similarities to MD, and from which cell lines were relatively easy to establish (Epstein and Barr, 1964; Epstein, 1970). In the present communication, the fine structure of two cell lines (Powell *et al.*, 1974) from MD lymphomata has been studied. Several similarities to, and some differences from, Burkitt's lymphoma-derived cell lines have been noted.

### MATERIALS AND METHODS

Ovarian lymphomata were collected from Houghton Poultry Research Station (HPRS) Rhode Island Red chickens experimentally infected at one day of age with a virulent strain of Marek's disease virus (HPRS-16). Dispersion of the tumours into single cell suspensions was by mechanical agitation in 1% trypsin. Cultures were initiated at 40°C at a concentration of $5 \times 10^6$ cells/ml in medium RPMI 1640 supplemented with 20% foetal calf serum and 10% tryptose phosphate broth. Cultures were refed by replacement of supernatant medium every 2 days for the first week of culture, taking care not to disturb the settled cells, and thereafter weekly until signs of growth were apparent. The actively growing cell lines were subcultured every 7 days.

Two lymphoblastoid cell lines, HPRS Line 1 and HPRS Line 2, have arisen from a total of 120 cultures initiated. Growth was detected 31 days and 92 days respectively after initiation. HPRS Line 1 has now been growing for 42 weeks and Line 2 for 24 weeks. Material for electron microscopy was taken from the cultures from time to time over several months. More samples from HPRS Line 1 have been examined to date than from HPRS Line 2.

For electron microscopy, the cells were pelleted, the culture fluid removed and the cells resuspended in 2% buffered glutaraldehyde for 30 min at 4°C. The fixed cells were then pelleted and the pellets cut into small cubes. The cubes were washed in buffer and were post-fixed for 1 h in cold 1% osmium tetroxide and dehydrated in increasing concentrations of ethyl alcohol. The cells were embedded in Araldite and thin sectioned with an LKB Ultratome III. The thin sections were stained with uranyl acetate and lead citrate and examined with a Philips EM 300 electron microscope.

For light microscopy, smears and cytocentrifuge (Cytospin: Shandon Elliott, Camberley, Surrey) preparations were stained with May–Grünwald–Giemsa stains.

### RESULTS

When examined in the living state with the light microscope, the cells varied from 5 to 12 $\mu$m in diameter, with a mean of 8 $\mu$m. In smears and cytocentrifuge preparations stained with May–Grünwald–Giemsa stains (Fig. 1) the cells had large nuclei and a rim of intensely staining basophilic cytoplasm containing vacuoles. Two nuclei were seen in some large cells but nucleoli were not observed.

When examined in the electron microscope, the cells were usually round (Fig. 2, 3) or sometimes oval in shape, although a few were elongated (Fig. 3). Some had small cytoplasmic processes (Fig. 3). The nuclei were large and usually round or oval and the nuclear membrane was often slightly indented (Fig. 2, 3). A thin rim of chromatin was usually arranged round the periphery of the nucleus and nucleoli were occasionally seen (Fig. 2, 3). About 3% of the cells showed projections of the nuclear envelope and many of the projections enclosed portions of cytoplasm (Fig. 3). Several mitochondria were present in the cytoplasm but the endoplasmic reticulum, which was usually rough surfaced, was sparse (Fig. 2, 3). A small Golgi apparatus was seen in some cells and centrioles, which were often accompanied by spindle tubules, were also observed. Small, often irregularly shaped osmiophilic bodies, which were probably composed of lipid, were present in most of the cells (Figs. 2, 3) although some cells contained many and others contained few of these organelles. Occasional vacuoles were present in the cyto-

FIG. 1.—Photomicrograph of a stained film of lymphoblasts of HPRS Line 1. The regular rounded cells have prominent nuclei and basophilic cytoplasm. Some large cells and binucleate cells (arrows) are present. May–Grünwald–Giemsa. ×2000.

FIG. 2.—Electron micrograph of a cell with a large nucleus containing a nucleolus. The cytoplasm contains ribosomes, mitochondria, a small lipid body (arrow), vacuoles (V) and sparse rough surfaced cisternae of endoplasmic reticulum (er). ×9000.

FIG. 3.—A round cell containing few cytoplasmic organelles except for abundant ribosomes. The adjacent cell is elongated and contains many lipid bodies (arrows). Both cells have chromatin arranged round the periphery of the nucleus, and small cytoplasmic processes (p) are present. A portion of a cell in mitosis (M) is also shown. ×8500. The inset is an enlarged portion of the elongated cell showing nuclear projections. ×18,000.

plasm (Fig. 2). Ribosomes, some of which were in the form of polyribosomes, were abundant throughout the cytoplasm (Fig. 2, 3). Crystalline structures (Fig. 4, 5) were occasionally seen in the cytoplasm of some cells. The cells with which these structures were associated were usually degenerate. Parallel arrays of annulate lamellae were present in the cytoplasm of a few cells (Fig. 6). Some cells contained many microtubules, about 17–25 nm in diameter, in their cytoplasm (Fig. 7).

A very small proportion of cells in HPRS Line 1 (less than 0·5%) contained particles resembling immature intranuclear herpesvirus capsids (Fig. 8). The particles measured about 90–95 nm in diameter and no enveloped forms or cytoplasmic particles were observed. No herpesvirus particles have been observed in Line 2, although fewer cells than from Line 1 have been examined to date.

The cultures were also examined for the presence of C-type particles. Such particles have not been observed.

DISCUSSION

The ultrastructural details of the avian cells examined in the present study closely resemble those of human lymphoblasts derived from Burkitt's lymphoma (Epstein and Achong, 1965, 1970). The main difference between the two cell lines is the presence of prominent nucleoli in the Burkitt's cells, both at the light and electron microscope levels (Epstein and Barr, 1964; Epstein and Achong, 1965, 1970) whereas this is not the case with the HPRS MD cell lines.

Similarities between the human and avian lines include the presence in both of abundant ribosomes, nuclear projections, annulate lamellae and only scanty endoplasmic reticulum and Golgi. Annulate lamellae are also a feature of cultured chick cells infected with strains of MD virus (Epstein *et al.*, 1968) although they have been seen in a number of other cell types.

Another similarity between lymphoblasts from Burkitt's lymphoma and the MD-derived cell lines is the presence in

FIG. 4, 5.—Crystalline structures in the cytoplasm of degenerating cells. The different appearance of these structures is probably due to differences in the plane of section. ×35,000.

FIG. 6.—Parallel arrays of annulate lamellae in the cytoplasm. Cross-sectional views (arrow) are also seen. ×47,000.

FIG. 7.—Portion of cytoplasm showing microtubules. ×51,000.

FIG. 8.—Portion of a cell containing immature intranuclear herpesvirus particles. ×22,000.

both of herpesvirus particles (Epstein *et al.*, 1964; Akiyama *et al.*, 1973; Akiyama and Kato, 1974). The viruses seen in the HPRS lines are presumably MD herpesvirus particles. It appears that infected cells are more abundant in the MD-derived cell lines of Akiyama (Akiyama *et al.*, 1973; Akiyama and Kato, 1974) than in the HPRS cell lines. Such variations are also known in Burkitt's lymphoma-derived lines where, although Epstein–Barr virus (EBV) production usually occurs in most lines, some lines are known in which virus production has not been detected by electron microscopy or immunofluorescence, although these lines are known to harbour the viral genome (Zur Hausen and Schulte-Holthausen, 1970). The EBV genome is considered to be present in most, if not all, cells of an infected Burkitt lymphoma-derived line (Miller, Stitt and Miller, 1970) and it has been suggested that EBV infection may be a prerequisite for the permanent growth of human lymphoblastoid cell lines *in vitro* (Nilsson *et al.*, 1972). A similar situation may also occur in Marek's disease, since recently, Nazerian (Nazerian *et al.*, 1973; Nazerian and Lee, 1975 in press) examined MD tumours and an MD-derived lymphoblastoid cell line by a molecular hybridization technique and detected an average of between 3 and 15 virus genome equivalents per cell in the tumours, and between 60 and 90 in the cell line. However, it is difficult to say whether all cells harbour a few genomes or whether a few cells contain many genomes. The greater number of viral genomes present in the cell line over those found in tumours probably reflects the heterogeneity of MD tumours.

A small proportion of cells in the HPRS lines have nuclear projections. Such projections are also a feature of some cells in MD tumours (Mladenov *et al.*, 1972; Doak, Munnell and Ragland, 1973; Frazier, 1974). The presence of nuclear projections has been reported in the lymphoid cells of other neoplasms including Burkitt's lymphoma (Achong and Epstein, 1966; Papadimitriou, 1966; Mollo and Stranignoni, 1967; Parker, Wakasa and Lukes, 1967; Miller *et al.*, 1969). Recent work (Weber *et al.*, 1973) suggests a relationship between nuclear projections in bovine peripheral blood lymphocytes and production of C-type virus particles in cultures of these cells. However, C-type particles were not observed in the HPRS lines, nor in those of Akiyama (Akiyama *et al.*, 1973; Akiyama and Kato, 1974). Nuclear projections have also been seen in apparently normal cells (Sebuwufu, 1966; Törö and Oláh, 1966; Huhn, 1967; Smith and O'Hara, 1967) including lymphocytes cultured with phytohaemagglutinin (Mollo and Stranignoni, 1967; Frazier, unpublished observations). However, they are rarely seen in the lymphoid organs of healthy chickens (Frazier, 1974). It would thus appear that although cells with nuclear projections could be neoplastic, they can be a feature of normal lymphoid cells although they tend to be associated with increased proliferation of the cells.

Cytoplasmic crystalline structures similar to those observed in the present study have been seen in other situations. They have been observed in cultured chick cells infected with strains of Marek's disease (Frazier, unpublished observations) and turkey herpesvirus (Nazerian *et al.*, 1971). They are also present in short-term cultured blood lymphocytes from birds with MD (Campbell and Woode, 1970) and it has been suggested that they represent abortive attempts to assemble virus in the cytoplasm. However, in the present study and that of Nazerian *et al.* (1971), the crystalline structures were usually associated with degenerating cells. Other work suggests that they are caused by ribosome crystallization (Maraldi and Barbieri, 1969; Barbieri *et al.*, 1970).

The ultrastructure of the HPRS cell lines not surprisingly resembles that of many of the cells in MD tumours. The tumours are composed of both thymus- and bursa-dependent cells although most are of thymic origin (Hudson and Payne, 1973; Rouse, Wells and Warner, 1973).

The MD-derived HPRS cell lines are also of thymic origin (Powell *et al.*, 1974). This identification is supported by the ultrastructural finding of abundant ribosomes and sparse endoplasmic reticulum in the cells. This and other work suggest that the development of lymphomata in Marek's disease is dependent upon malignant transformation of thymus-dependent lymphocytes by MD virus.

We thank Mrs Carolyn Broadley, Mr A. W. Kidd and Miss Brenda Parish for technical assistance and Mrs Elizabeth Sefton for secretarial assistance.

REFERENCES

ACHONG, B. G. & EPSTEIN, M. A. (1966) Fine Structure of the Burkitt Tumour. *J. natn. Cancer Inst.*, **36**, 877.

AKIYAMA, Y. & KATO, S. (1974) Two Cell Lines from Lymphomas of Marek's Disease. *Biken's J.* **17**, 105.

AKIYAMA, Y., KATO, S. & IWA, N. (1973) Continuous Cell Culture from Lymphoma of Marek's Disease. *Biken's J.*, **16**, 177.

BARBIERI, M., SIMONELLI, L., SIMONI, P. & MARALDI, N. M. (1970) Ribosome Crystallization II Ultrastructural Study on Nuclear and Cytoplasmic Ribosome Crystallization in Hypothermic Cell Cultures. *J. submicrosc. Cytol.*, **2**, 33.

CAMPBELL, J. G. & WOODE, G. N. (1970) Demonstration of a Herpes-type Virus in Short-term Cultured Blood Lymphocytes Associated with Marek's Disease. *J. med. Microbiol.*, **3**, 463.

DOAK, R. L., MUNNELL, J. F. & RAGLAND, W. L. (1973) Ultrastructure of Tumor Cells in Marek's Disease Virus-infected Chickens. *Am. J. vet. Res.*, **34**, 1063.

EPSTEIN, M. A. (1970) Long-term Tissue Culture of Burkitt Lymphoma Cells. In *Burkitt's Lymphoma*. Ed. D. P. Burkitt and D. H. Wright. Edinburgh & London: E. & S. Livingstone. p. 148.

EPSTEIN, M. A., ACHONG, B. G. & BARR, Y. M. (1964) Virus Particles in Cultured Lymphoblasts from Burkitt's Lymphoma. *Lancet*, i, 702.

EPSTEIN, M. A. & ACHONG, B. G. (1965) Fine Structural Organization of Human Lymphoblasts of a Tissue Culture Strain (EB1) from Burkitt's Lymphoma. *J. natn. Cancer Inst.*, **34**, 241.

EPSTEIN, M. A. & ACHONG, B. G. (1970) The Fine Structure of Cultured Burkitt Lymphoblasts of Established *in vitro* Strains. In *Burkitt's Lymphoma*. Ed. D. P. Burkitt and D. H. Wright Edinburgh & London: E. & S. Livingstone p. 118.

EPSTEIN, M. A., ACHONG, B. G., CHURCHILL, A. E. & BIGGS, P. M. (1968) Structure and Development of the Herpes-type Virus of Marek's Disease. *J. natn. Cancer Inst.*, **41**, 805.

EPSTEIN, M. A. & BARR, Y. M. (1964) Cultivation *in vitro* of Human Lymphoblasts from Burkitt's Malignant Lymphoma. *Lancet*, i, 252.

FRAZIER, J. A. (1974) Ultrastructure of Lymphoid Tissue from Chicks Infected with Marek's Disease Virus. *J. natn. Cancer Inst.*, **52**, 829.

HUDSON, L. & PAYNE, L. N. (1973) An Analysis of the T and B Cells of Marek's Disease Lymphomas of the Chicken. *Nature, New Biol.*, **241**, 52.

HUHN, D. (1967) Nuclear Pockets in Normal Monocytes. *Nature, Lond.*, **216**, 1240.

KLEIN, G. (1972) A Summing Up. In *Oncogenesis and Herpeseviruses*. Ed. P. M. Biggs, G. de Thé and L. N. Payne. Lyon: International Agency for Research on Cancer. p. 501.

MARALDI, N. M. & BARBIERI, M. (1969) Ribosome Crystallization I Study on Electron Microscope of Ribosome Crystallization during Chick Embryo Development. *J. submicrosc. Cytol.*, **1**, 159.

MILLER, J. M., MILLER, L. D., GILLETTE, K. G. & OLSON, C. (1969) Incidence of Lymphocytic Nuclear Projections in Bovine Lymphosarcoma. *J. natn. Cancer Inst.*, **43**, 719.

MILLER, M. H., STITT, D. & MILLER, G. (1970) Epstein–Barr Viral Antigen in Single Cell Clones of Two Human Leukocytic Lines. *J. Virol.*, **6**, 699.

MLADENOV, Z., BOZHKOV, S., TODOROV, T. G. & KIREV, T. (1972) Pathomorphological and Ultrastructural Studies on Marek's Disease in Fowls. *Bull. Inst. gen. comp. Pathol.*, **14**, 73.

MOLLO, F. & STRANIGNONI, A. (1967) Nuclear Projections in Blood and Lymph Node Cells of Human Leukaemias and Hodgkin's Disease and in Lymphocytes Cultured with Phytohaemagglutinin. *Br. J. Cancer*, **21**, 519.

NAZERIAN, K. & LEE, L. F. (1975) Deoxyribonucleic Acid of Marek's Disease Virus in a Lymphoblastoid Cell Line from Marek's Disease Virus Tumors. *J. gen. Virol.* In the press.

NAZERIAN, K., LEE, L. F., WITTER, R. L. & BURMESTER, B. R. (1971) Ultrastructural Studies of a Herpesvirus of Turkeys Antigenically Related to Marek's Disease Virus. *Virology*, **43**, 442.

NAZERIAN, K., LINDAHL, T., KLEIN, G. & LEE, L. F. (1973) Deoxyribonucleic Acid of Marek's Disease Virus in Virus-induced Tumours. *J. Virol.*, **12**, 841.

NILSSON, K., KLEIN, G., HENLE, G. & HENLE, W. (1972) The Role of EBV in the Establishment of Lymphoblastoid Cell Lines from Adult and Foetal Lymphoid Tissue. In *Oncogenesis and Herpesviruses*. Ed. P. M. Biggs, G. de Thé and L. N. Payne. Lyon: International Agency for Research on Cancer. p. 285.

PAPADIMITRIOU, J. M. (1966) Electron Microscopic Findings of a Murine Lymphoma Associated with Reovirus Type 3 Infection. *Proc. Soc. exp. Biol. Med.*, **121**, 93.

PARKER, J. W., WAKASA, H. & LUKES, R. J. (1967) Canine and Burkitt's Lymphoma. *Lancet*, i, 214.

POWELL, P. C., PAYNE, L. N., FRAZIER, J. A. & RENNIE, M. (1974) T Lymphoblastoid Cell Lines from Marek's Disease Lymphomas. *Nature, Lond.*, **251**, 79.

ROUSE, B. T., WELLS, R. J. & WARNER, N. L. (1973) Proportion of T and B Lymphocytes in Lesions of Marek's Disease: Theoretical Implications for Pathogenesis. *J. Immun.*, **110**, 534.

SEBUWUFU, P. H. (1966) Nuclear Blebs in the Human Foetal Thymus. *Nature, Lond.*, **212**, 1382.

SMITH, G. F. & O'HARA, P. T. (1967) Nuclear

Pockets in Normal Leucocytes. *Nature, Lond.*, **215**, 773.

TÖRÖ, I. & OLÁH, I. (1966) Nuclear Blebs in the Cells of the Guinea Pig Thymus. *Nature, Lond.*, **212**, 315.

WEBER, A., FAHNING, M., HAMMER, R. H. & JESSEN, C. (1973) Relationship between Nuclear Pockets in Bovine Peripheral Blood Lymphocytes and C-type Virus Particles in Cultures of these Cells. *J. natn. Cancer Inst.*, **51**, 81.

ZUR HAUSEN, H. & SCHULTE-HOLTHAUSEN, H. (1970) Presence of EB Virus Nucleic Acid Homology in a Virus-free Line of Burkitt Tumour Cells. *Nature, Lond.*, **227**, 245.